# Correlation Analyses of Computed Tomography and Magnetic Resonance Imaging for Calculation of Prostate Volume in Colorectal Cancer Patients with Voiding Problems Who Cannot Have Transrectal Ultrasonography

**DOI:** 10.1155/2019/7029450

**Published:** 2019-03-31

**Authors:** Sung Han Kim, Boram Park, Whi-An Kwon, Jae Young Joung, Ho Kyung Seo, Jinsoo Chung, Kang Hyun Lee

**Affiliations:** ^1^Department of Urology, Urological Cancer Center, Research Institute and Hospital of National Cancer Center, Goyang, Republic of Korea; ^2^Biometrics Research Branch, Division of Cancer Epidemiology and Prevention, Research Institute and Hospital of National Cancer Center, Goyang, Republic of Korea

## Abstract

**Objective:**

To evaluate the value of computed tomography (CT) and magnetic resonance imaging (MRI) in determining total prostate volume (TPV) for patients with colorectal cancer, as an alternative to transrectal ultrasonography (TRUS) of the prostate when TRUS is not an option.

**Methods:**

We retrospectively evaluated the medical records of 122 male cancer patients who were referred to our urology department between 2014 and 2016 for voiding problems. They underwent colorectal surgery within 3 months; we estimated the correlations of the TPV measurements made using CT, MRI, and TRUS. A total of 122 TRUS, 88 MRI, and 34 CT images were reviewed repeatedly, twice by 2 independent urologists within 1 month after the initial evaluation. The correlations were statistically evaluated using a Bland-Altman plot and Spearman and Pearson correlation analyses.

**Results:**

Overall median age was 70.5 years and the median TPV, as measured using TRUS, CT, and MRI, was 33.2, 43.4, and 30.1 mL, respectively. There was a good correlation in TPV measured with CT (coefficient >0.7) and MRI (>0.8). There was not a good correlation between TRUS and preoperative and postoperative CT/MRI; preoperative CT/MRI had a higher correlation (>0.7) than postoperative CT/MRI (>0.8). When stratified by prostate volume, preoperative CT (>0.58-0.59) correlated better for <30 mL and preoperative MRI (0.70-0.75) correlated better for ≥30 mL.

**Conclusions:**

The study showed that preoperative MRI had the best correlation with TRUS, especially in prostates ≥30 mL despite overestimations in CT and MRI measurements compared with TRUS.

## 1. Introduction

The increasing lifespan and high-calorie intake of the westernized lifestyle have contributed to a rapid increase in the incidence of patients with colorectal cancer (CRC) in Asia [1]. CRC has been reported as the third most common cancer, two times more predominant in men in 2012, and with a 30-40% higher rate of overall survival and mortality than in women [2, 3]. An increasing number of elderly patients with CRC undergo curative surgery to achieve oncologic control as the lifespan expectancy has been prolonged [2–4].

As for male patients with CRC >65 years old, diverse postoperative complications and impaired quality of life have been frequently encountered, such as voiding dysfunction [1]. Bladder dysfunction following colorectal surgery is most commonly related to extirpative procedures in the region of the autonomic pelvic plexus with an incidence rate of 15-50% after surgery [5, 6]. Although the most frequent cause of bladder dysfunction after colorectal surgery is the disruption of the autonomic nerve plexus, up to 40% of elderly male patients with an intact autonomic nerve plexus have predisposing lower urinary tract outlet abnormalities, such as benign prostatic hyperplasia (BPH) [1].

To differentiate patients with bladder dysfunctional due to lower urinary tract obstruction from those with intraoperative autonomic nerve plexus injury, measurement of total prostate volume (TPV) and its degree of obstruction in the lower urinary tract is important. It is typically measured using transrectal ultrasonography of prostate (TRUS) via the anus as well as cystoscopic evaluation [6–8]. Patients who underwent colorectal surgery can only have cystoscopy, as they cannot undergo TRUS until 3 months after surgery [8]. An alternative imaging modality other than TRUS would be needed to evaluate the TPV after colorectal surgery. The therapeutic effectiveness of the bladder dysfunction treatment will be improved if the TPV is correctly estimated and lower urinary tract obstruction can be ruled out using TRUS and other evaluating tools.

Under the circumstances in which TRUS cannot be used, computed tomography (CT) and magnetic resonance imaging (MRI) are other possible measuring tools for imaging of the prostatic anatomy because they are widely used imaging modalities for CRC, preoperatively and postoperatively. Therefore, this study investigated the correlation and reliability of prostate volume measurements by CT or MRI compared with TRUS in patients with CRC who underwent colorectal surgery.

## 2. Materials and Methods

### 2.1. Ethical Statement

All study protocols were conducted according to the ethical guidelines of the “World Medical Association Declaration of Helsinki-Ethical Principles for Medical Research Involving Human Subjects.” This study was approved by the Institutional Review Board of the Research Institute and Hospital National Cancer Center (IRB No. NCC2016-0277). The requirement for informed consent from all of the patients was waived by the IRB.

### 2.2. Subjects and Clinical Parameters

From January 2014 to December 2016, 122 patients underwent colorectal surgery and had preoperative TRUS and either CT or MRI or both preoperatively and postoperatively under the discretion of the respective surgeon and according to the type of colorectal cancers. A total of 37 patients had enhanced CT images and 88 had contrast-enhanced MRI images within a 3-month interval after colorectal surgery. Patients were excluded for the following: urethral catheters, diagnosed as having prostate cancer, undergoing previous prostatectomy, neoadjuvant/adjuvant history of chemotherapy, or not having a TRUS-measured prostate volume. Age, height, weight, underlying diseases such as BPH, diabetes mellitus, hypertension, cerebrovascular disease, and others, and radiologic imaging were collected retrospectively.

### 2.3. Total Prostate Volume (TPV) Measurement

All TRUS procedures and urologic imaging interpretations were performed by a uroradiologist with 15 years of experience. TRUS-measured TPV was calculated by applying the ellipsoid formula: *π*/6 × [width (cm)] × [length (cm)] × [height (cm)]. We considered the TRUS-measured prostate as the true volume of the prostate.

The prostate volume measurement was independently measured by two blinded urologists with 7 years of experience after reviewing CT/MR images (JK Kim and YS Suh). To ensure standardization of measurements, an orientation was conducted by the investigator before the images were reviewed. All the participants were blinded to the TRUS-measured prostate volume results. The length and width of the prostate were measured in axial views, and the height was measured in sagittal views. Prostate volumes measured by CT and MRI were calculated by using the ellipsoid formula: 0.52 × [width (cm)] × [length (cm)] × [height (cm)].

### 2.4. Comparison of CT/MRI and TRUS for TPV Measurement

TPVs were calculated to evaluate the correlation between those measured by CT/MRI and those measured by TRUS. Bland-Altman plots with multiple measurements per subject were performed to compare the two methods. To investigate the effect of prostatic size on the accuracy of the measurements, prostate volumes according to TRUS were classified into 2 categories: ≤30 mL and >30 mL.

### 2.5. The Reliability of CT Measurement: Inter- and Intrapersonal Variation Test-Retest

To determine inter- and intraobserver reliability tests, the results of the 2 independent interpreters were compared for both the test and retest using an intraclass correlation coefficient (ICC) (Supplementary Table 1). To evaluate the test-retest reliability, the same images were reviewed after 1 month, with the participants blinded to the results of the previous measurements.

### 2.6. Statistical Analyses

The baseline characteristics were summarized as median (range; minimum-maximum) for continuous variables and frequency (percentage) for categorical variables. Pearson's correlation coefficients were calculated to investigate how TRUS measurements correlated with CT and MRI measurements of TPV. Bland-Altman plots were also used to examine agreement between the CT and MRI measurements and the TRUS value. The closer the mean difference to zero, the better the agreement between the measures. TRUS-measured TPV tends to be overestimated (i.e., the mean difference is greater than zero) or underestimated (i.e., the mean difference is less than zero) compared with TPV measured by CT or MRI. The statistical limits (lower and upper) of agreement using the mean and standard deviation of the differences were presented with the mean difference. The MRI and CT images were reviewed twice by two urologists, as described above. The ICC was used to assess how consistent the estimated prostate volumes are with each other. For all analyses, a p-value less than 0.05 was considered statistically significant and statistical analyses were performed using SAS software, version 9.4 (SAS Institute Inc., Cary, NC, USA), and R software, version 3.3.3 (R Project for Statistical Computing).

## 3. Results

The median age of the patients was 70.5 years (range, 40.0-90.0 years). The concomitant diseases were as follows: benign prostatic hyperplasia (20 patients, 16.4%), hypertension (48 patients, 39.3%), diabetes (28 patients, 23.0%), and cerebrovascular disease (10 patients, 8.2%). Baseline characteristics, including the type of surgery, preoperative voiding information, and postoperative voiding information are described in Table 1. Only 8 (6.6%) patients had a preoperative history of voiding problems assessed via the voiding symptom questionnaire.

The median TRUS-TPV for all 122 patients was 25.0 mL (range, 7.0-191.0 mL). The median (range) of the preoperative CT volumes measured by the first and second urologists was 43.4 mL (10.2-131.5 mL) and 42.4 mL (11.0-123.1 mL), respectively. The median (range) of the postoperative CT volumes as measured by the first and second urologists was 40.6 mL (30.3-55.0 mL) and 37.3 mL (31.4-53.6 mL), respectively. The median (range) of the preoperative MRI volumes measured by the first and second urologists was 29.7 mL (15.1-108.7 mL) and 33.6 mL (15.8-101.9 mL), respectively. The median (range) of the postoperative MRI volumes measured by the first and second urologists was 33.0 mL (11.7-67.1 mL) and 33.1 mL (15.2-75.1 mL), respectively (Table 1).

The ICCs between the two urologists were all above 0.9. Therefore, based on the ICCs and Bland-Altman plots, the agreement between the two urologists for the pre- and postoperative Ct and MRI measurements was excellent (Supplementary Table 1, Supplementary Figure 1).

**Figure 1:**
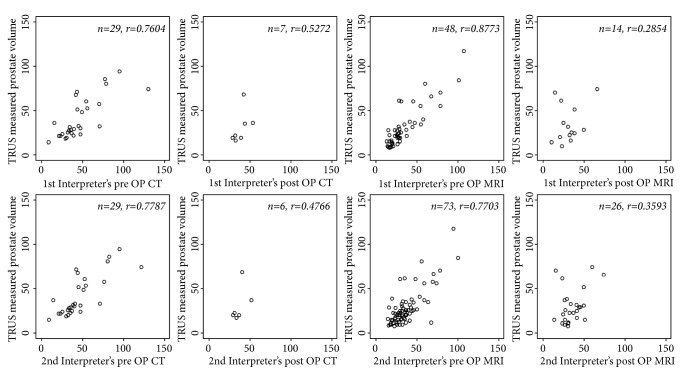
Scatter plot between TRUS total prostate volume and pre- and post-CT/MRI volume.

Pearson's correlation coefficients were assessed to evaluate whether CT and MRI could replace TRUS when TRUS is not an option. Pre- and postoperative CT and MRI images were reviewed, but for many patients the postoperative images were not measured. Pearson's correlation coefficients for the preoperative measurements were 0.7604 and 0.7787 (for the first and second urologists, respectively) between the volumes measured by TRUS and CT, and 0.8773 and 0.7703 (for the first and second urologists, respectively) between the TRUS and MRI volumes. The postoperative Pearson's correlation coefficients between TRUS and CT volume were 0.5272 and 0.4766 (for the first and second urologists, respectively), and 0.2854 and 0.3593 (for the first and second urologists, respectively) between TRUS and MRI volume. The preoperative correlations between TRUS and CT and MRI were higher than those for the postoperative measurements (Table 2, Figure 1).

**Figure 2:**
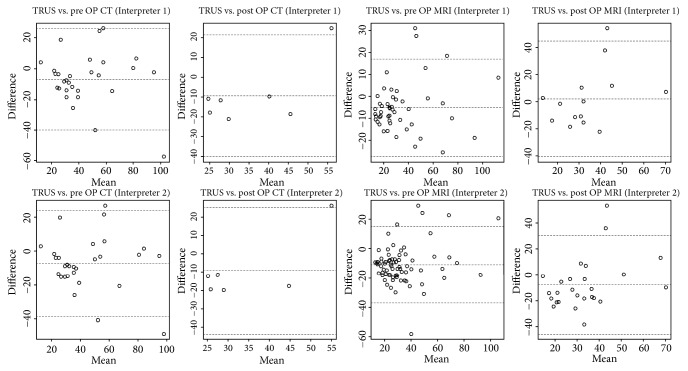
Bland-Altman plot to compare prostate volume according to CT and TRUS.

The agreements between CT, MRI, and TRUS were also confirmed using Bland-Altman plots (Figure 2). For the first urologist, the mean (lower, upper limit) differences in the preoperative CT and MRI measurements were -7.17 (-40.33, 25.99) and -5.21(-27.31, 16.9) and for postoperative measurements were -9.73 (-40.29, 20.82) and 1.74 (-41.07, 44.54), respectively. For the second urologist, the mean differences for the preoperative CT and MRI measurements were -7.29 (-38.78, 24.2) and -10.71 (-36.75, 15.33) and for postoperative measurements were -9.23 (-43.7, 25.23) and -7.79 (-45.97, 30.39), respectively. Overall, the TPV measured by CT and MRI tended to be overestimated compared with TRUS.

Subgroup analyses were performed by dividing the TPV measured by TRUS into two groups: <30 mL and ≥30 mL. Although the sample size was reduced when divided into two groups, the correlation between the CT, MRI, and TRUS measurements was still higher preoperatively. In the TPV <30 mL subgroup, TPV measured by CT correlated better with TRUS than TPV measured by MRI. However, TPV measured by MRI had a higher correlation with TRUS than TPV measured by CT when the TPV size was ≥30 mL (Table 3).

## 4. Discussion 

The type of voiding dysfunction after CRC depends on the surgical procedure. The highest incidence of voiding dysfunction (approximately 50%) was reported for abdominoperineal resection and an incidence of 15-20% is reported for low anterior resection [9]. The main cause of postoperative voiding dysfunction is autonomic nerve plexus disruption that requires postoperative interventional management, such as intermittent catheterization, indwelling urethral catheterization, or suprapubic cystostomy.

In addition to the autonomic nerve disruption causes, obstructive lower urinary tract-related voiding symptoms are usually associated with enlargement of the prostate in elderly male patients (60-year-old with 60%, 70s with 70%, and 80s with 80%). These patients do not need any postoperative interventional management but benefit from medical therapies for BPH such as alpha-blockers and surgery to reduce a large TPV [10–13]. Accurate prostate volume determination is useful and critical for those patients with BPH.

Although TPV is not correlated with symptom severity [14], patients with a prostate volume of >40 mL have significant relief of voiding symptoms with a combination therapy of alpha-blockers and 5-alpha-reductase inhibitors; a single alpha-blocker is effective for patients with prostate volume <40 gm [15]. Those patients with a very large prostate volume (> 80 gm) are indicated for surgical prostatectomy, such as Holmium laser enucleation of the prostate or transurethral prostatectomy, rather than medical therapy [16].

TRUS is the standard modality for prostate volume measurement and is a versatile modality with an easy accessibility. However, TRUS cannot be used under certain conditions, such as when anal strictures are present, or for 2 – 3 months after CRC surgery [1]. In such cases, CT or MRI might be an alternative imaging option to determine prostate volume. However, this study found that neither CT nor MRI have successfully demonstrated a high correlation with the TPV as measured by TRUS in pre- or postoperative settings. This is likely because of the small number of cases and the retrospective design. However, some important clinical findings were observed that suggest that preoperative MRI had the highest correlation with the TPV measurement by TRUS, especially for TPV >30 mL. A large-scale, prospective study would be needed to evaluate the feasibility of preoperative MRI as an alternative modality to TRUS in patients who have undergone CRC surgery.

Many researchers have already tried to define the best alternative modality to TRUS for prostate measurement by analyzing the correlation between CT or MRI and TRUS [17–19]. In a cohort of patients with prostate cancer, Hoffelt et al. showed that CT overestimated the prostate volume as compared with TRUS by up to 50%. Park et al. also compared prostate volume measured by prebrachytherapy CT or MRI with prostate volume by TRUS in patients with prostate cancer, including patients receiving neoadjuvant hormone therapy [20]. They showed that the prostate volume was roughly overestimated by 1.36 times with CT and by 1.33 times with MRI, with a mean difference of 9.05 mL in CT and 6.84 mL in MRI. Therefore, MRI was more closely correlated with the TRUS, similar to the finding in this study. Kang et al. overestimated the prostate volume by 8.4% compared with TRUS in patients with lower urinary tract symptoms [21].

Few studies have reported the correlation of prostate volume in patients without prostate cancer, and no reports have been made for patients with CRC. It has been suggested in previous studies that CT and MRI are inaccurate for prostate imaging, similar to this study in which we identified a weak correlation coefficient of less than 0.8, except for preoperative MRI (correlation coefficient, >0.8) (Table 2) [22, 23].

However, TRUS is a subjective and operator-dependent modality influenced by the size of the prostate gland. The actual gland size can be over- or underestimated, according to the size of the pathological specimen [24]. Bienz et al. have shown that TRUS underestimates the prostate volume when the prostate is smaller in size, and overestimates the volume when the prostate is larger; however, the measurements were more accurate for larger prostates, similar to the findings in this study (Table 3) [24]. We found that, when stratified by a prostate size of 30 mL, TRUS and CT or MRI did not correlate well (correlation coefficient 0.3-05 for prostate size < 30 mL, Table 2). The CT and MRI had a better correlating power (0.5-0.7) for prostate size ≥30 mL (Table 2).

This overestimated TPV as measured by CT or MRI is explained by an inherent error rate of the CT ellipsoid formula and the low soft tissue resolution around the prostate and the intraprostatic anatomy [25]. To enhance the exact volume calculation, step-section planimetry is regarded to be a more accurate method for the measurement of prostate volume [26–29]. However, it is more time consuming than the easily usable ellipsoid formula and requires the use of special equipment that it is not useful in a clinical setting. Eri et al. have shown that the simple ellipsoid formula was only marginally inferior to step-section planimetry [30]. Another accurate modality suggested by Jeong et al. was planimetry using the 3-dimensional reconstruction method in MRI [26]. However, it is more expensive, and MRI is usually not indicated for purposes such as routine check-ups. In this study, the prostate volume on axial and coronal views was used with the ellipsoid formula of width x height x length x *π*/6, because the axial and coronal views, or only the axial view, may be the only CT/MRI images available for calculation in real clinical settings.

The postoperative prostate measurement was smaller than the preoperative TPV measurement and a wider range of different volume measurements was detected using CT in this study (Table 1). One of the possible explanations is that removal of the mass by CRC surgery might affect the shape of the prostate anatomically, such that the prostate had been compressed and deformed by the colorectal mass. Another explanation might be that the inflammation and edema in the periprostatic tissue and the prostate resulted in preoperative overestimation of the TPV. After removal of the cancer and postoperative antibiotic management, the inflammatory and edematous prostate decreased to its normal size and repositioned to its normal anatomic shape to result in a decreased prostate volume measurement. Lastly, removal of periprostatic tissue by intraoperative adhesiolysis during CRC surgery causes prostatic atrophy or disappearance of periprostatic overenhancement. Adhesiolysis of the perimesorectal to periprostatic tissue and the prostatic capsule are needed to achieve free movability of the colon for the anastomosis to the anus in transanal total mesorectal resection where the periprostatic tissue was removed and appeared postoperatively.

This study has a few limitations, such as a retrospective design with a small number of cases and different types of colorectal procedures, as well as use of the ellipsoid equation to calculate volume. The different types of colorectal surgery might have an influence on the different rates and types of voiding problems, postoperatively. However, this study gave important clinical clues for the treatment of bladder dysfunction in elderly male patients with CRC after surgery. The necessity and importance of preoperative assessment of voiding suggests that clinicians need to plan the postoperative management of patients with voiding dysfunction by differentiating obstructive lower urinary tract disease from other etiologies. A simple voiding questionnaire, uroflowmetry, and serum prostatic specific antigen test are enough to predict patients with a high risk of voiding problems at outpatient clinics and TRUS can then be performed preoperatively. However, further prospective-designed studies with large numbers of patients according to the type of colorectal surgery will be needed for the evaluation of the appropriate imaging modalities for TPV measurement and to determine the efficacy of preoperative voiding assessments for postoperative obstructive lower urinary tract disease.

## 5. Conclusions

As patients undergoing CRC are predominantly elderly men, preoperative MRI is the best alternative modality for TPV measurement, even though it overestimates it when the TPV is > 30 mL, for these patients who cannot undergo a TRUS assessment.

## Figures and Tables

**Table 1:** Baseline demographics.

Parameter	N(%) or median (range)
Age (years)	70.5 (40.0-90.0)
Benign prostatic hyperplasia, n (%)	20 (16.4)
Hypertension, n (%)	48 (39.3)
Diabetes, n (%)	28 (23.0)
Cerebrovascular disease, n(%)	10 (8.2)
Cardiovascular disease, n(%)	4 (3.3)
Others, n (%)+	21 (12.2)
Preoperative IPSS score, total/QoL^++^	13.5 (0-35)/ 1 (1-6)
ASA score, 1/2/3	2/101/3 (1.9/95.3/2.8)
Preoperative voiding problem	8 (6.6)
Type of colorectal surgery	
Low anterior resection	68(55.7)
Hatmann (Proctosigmoidectomy)	2(1.6)
Miles operation (Abdominoperineal resection)	5(4.1)
Anterior resection of rectum	15(12.3)
Transanal total mesorectal excision	3 (2.5)
Subtotal- or hemi-colectomy	15 (12.3)
Others	14 (11.5)
Postoperative IPSS*∗*	
Symptom Score	19 (10-35)
Quality of Life score	7 (3-7)
Postoperative uroflowmetry	
Maximal flow rate (ml/hr)	49 (9.1-49.1)
Residual urine (cc)	158.5 (0-600)
Time interval between CT/MRI and TRUS (days)	25.0 (12-30)
TRUS-TPV (cc)	25.0 (7.0-191.0)
Pre-CT volume, 1st / 2nd person	43.4 (10.2-131.5) / 42.4 (11.0-123.1)
Post CT volume, 1st / 2nd person	40.6 (30.3-55.0) / 37.3 (31.4-53.6)
Pre-MRI volume, 1st / 2nd person	29.7 (15.1-108.7) / 33.6 (15.8-101.9)
Post MRI volume, 1st / 2nd person	33.0 (11.7-67.1) / 33.1 (15.2-75.1)

+, Others included hepatitis, hyperlipidemia, chronic pulmonary obstructive disease, and asthma;++, only 8 patients completed preoperative IPSS questionnaires; *∗*, only 72 and 77 patients completed postoperative IPSS questionnaires and uroflowmetry, respectively; PSA, prostate specific antigen; IPSS, International Prostatic Symptom Score questionnaire; ASA, American Society of Anesthesiologists score; TRUS, transrectal ultrasonography of prostate; CT, computed tomography; MRI, magnetic resonance imaging

**Table 2:** Pearson correlation between TRUS total prostate volume and pre- and post-CT/MRI volume.

		N	Pearson
			Correlation
TRUS total prostate volume	1st person Pre-CT volume	29	0.7604
TRUS total prostate volume	1st person Post CT volume	7	0.5272
TRUS total prostate volume	1st person Pre-MRI volume	48	0.8773
TRUS total prostate volume	1st person Post MRI volume	14	0.2854

TRUS total prostate volume	2nd person Pre-CT volume	29	0.7787
TRUS total prostate volume	2nd person Post CT volume	6	0.4766
TRUS total prostate volume	2nd person Pre-MRI volume	73	0.7703
TRUS total prostate volume	2nd person Post MRI volume	26	0.3593

**Table 3:** Pearson correlation between TRUS total prostate volume and pre- and post-CT/MRI volume according to the size of prostate volume with a cut-off of 30 gm.

		<30 gm		≥30 gm	
		N	Pearson Correlation	N	Pearson Correlation
TRUS total prostate volume	1st person Pre-CT volume	13	0.6645	16	0.6142
TRUS total prostate volume	1st person Post CT volume	4	-0.0275	3	-0.5782
TRUS total prostate volume	1st person Pre-MRI volume	31	0.5550	17	0.7382
TRUS total prostate volume	1st person Post MRI volume	8	0.7599	6	0.2966

TRUS total prostate volume	2nd person Pre-CT volume	13	0.6532	16	0.6389
TRUS total prostate volume	2nd person Post CT volume	4	-0.2757	2	-1.0000
TRUS total prostate volume	2nd person Pre-MRI volume	52	0.3192	21	0.6925
TRUS total prostate volume	2nd person Post MRI volume	16	0.4399	10	0.1941

## Data Availability

The datasets used and/or analysed during the current study are available from the corresponding author (Kang Hyun Lee, uroonco@ncc.re.kr; 5@ncc.re.kr) on reasonable request. The IRB and ethical committee of the National Cancer Center (in Korea) will review the requests because of the patients' information. After the approval of the committee with confirmation of the reasonable requests, the dataset will be freely available. The other contact e-mail besides the corresponding author's e-mail is irb@ncc.re.kr.

## Supplementary Materials

Supplementary 1 Supplementary Table 1: (A)interpersonal variation test (intraclass correlation coefficient between 1st person and 2nd person).

Supplementary 2 Supplementary Figure 1: Interpreter 1 vs. 2.
