# Supplementary material for: Correlation Analyses of Computed Tomography and Magnetic Resonance Imaging for Calculation of Prostate Volume in Colorectal Cancer Patients with Voiding Problems Who Cannot Have Transrectal Ultrasonography
Source: Biomed Res Int. 2019 Mar 31;2019:7029450. doi: 10.1155/2019/7029450 (PMC6462342; doi:10.1155/2019/7029450)
Supplement: Supplementary 1 — Supplementary Table 1: (A)interpersonal variation test (intraclass correlation coefficient between 1st person and 2nd person). [file 7029450.f1.docx]

Supplementary table 1. (A)Interpersonal variation test (Intraclass correlation coefficient between 1^st^ person and 2^nd^ person)

|  | median (min-max) | | N | ICC | lower | upper |
| --- | --- | --- | --- | --- | --- | --- |
|  | 1st person | 2nd person |  |  |  |  |
| CT volume | 43.39 (10.25 - 131.53) | 42.08 (10.99 - 123.10) | 33 | 0.9939 | 0.9877 | 0.9970 |
| Pre-OP CT | 43.39 (10.25 - 131.53) | 42.40 (10.99 - 123.10) | 29 | 0.9941 | 0.9874 | 0.9972 |
| Post OP CT | 37.37 (30.28 - 55.00) | 37.30 (31.37 - 53.64) | 6 | 0.9865 | 0.9071 | 0.9981 |
| MRI volume | 30.10 (11.74 - 108.69) | 32.63 (15.16 - 101.89) | 56 | 0.9769 | 0.9610 | 0.9864 |
| Pre-OP MRI | 29.69 (15.07 - 108.69) | 31.87 (15.83 - 101.89) | 48 | 0.9829 | 0.9697 | 0.9904 |
| Post OP MRI | 32.95 (11.74 - 67.12) | 31.95 (15.16 - 61.02) | 14 | 0.9306 | 0.7987 | 0.9772 |
