# Supplementary figures and images for: Correlation Analyses of Computed Tomography and Magnetic Resonance Imaging for Calculation of Prostate Volume in Colorectal Cancer Patients with Voiding Problems Who Cannot Have Transrectal Ultrasonography
Source: Biomed Res Int. 2019 Mar 31;2019:7029450. doi: 10.1155/2019/7029450 (PMC6462342; doi:10.1155/2019/7029450)

Supplementary Figure 1.


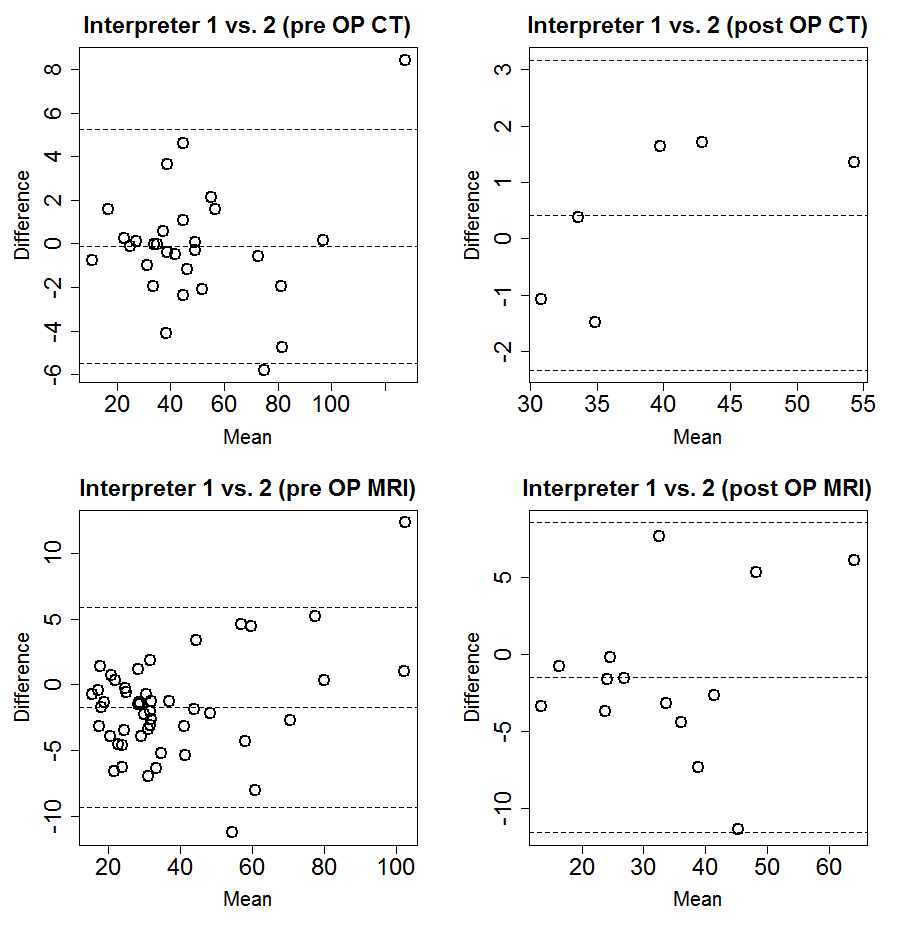

Supplement: Supplementary 2 — Supplementary Figure 1: Interpreter 1 vs. 2. [file 7029450.f2.docx]
